# A rare case of de novo 20p12.3 microdeletion syndrome in a nine-year-old female: case report and literature review

**DOI:** 10.3389/fgene.2025.1669947

**Published:** 2025-11-06

**Authors:** Hanan Aljedani, Yousef Faden, Manar Alghamdi, Alshaimaa Alzahrani, Aiman Shawli, Reem Albakistani

**Affiliations:** 1 College of Medicine, King Saud Bin Abdulaziz University for Health Sciences, Jeddah, Saudi Arabia; 2 King Abdullah International Medical Research Center, Jeddah, Saudi Arabia; 3 Genetics and Precision Medicine Department, King Abdullah Specialist Children Hospital, Jeddah, Saudi Arabia; 4 Pediatrics Department, King Abdullah Specialist Children Hospital, Jeddah, Saudi Arabia

**Keywords:** 20p12.3 microdeletion syndrome, growth faltering, short stature, seizure disorder, *de novo* gene deletion

## Abstract

Chromosomal deletion syndromes are common worldwide. However, one rare condition that distinguishes a limited number of reported cases and variable phenotypes is 20p12.3 microdeletion syndrome. This case report describes a nine-year-old girl diagnosed with 20p12.3 microdeletion syndrome. Genetic testing revealed a deletion spanning 3.5 Mb and containing 31 genes. The patient presented with a range of clinical manifestations, including growth faltering, short stature, controlled seizure disorder, dysmorphic features, and metabolic disturbances. Regarding dysmorphic features, she presented with malar hypoplasia, a high arched palate, microstomia, long philtrum, proptosis, and retrognathia. Metabolic disturbances were primarily manifested as episodes of hypoglycemia with a high anion gap metabolic acidosis. Despite receiving growth hormone therapy as management for short stature, the patient exhibited low levels of insulin-like growth factor 1 (IGF1). This case report adds to the limited body of knowledge regarding 20p12.3 microdeletion syndrome.

## Introduction

Chromosomal deletion syndromes are genetic disorders characterized by deletions of parts of chromosomes that cannot be detected using conventional karyotyping techniques (Antkowiak et al., 2023). They manifest with diverse symptoms, including intellectual disability, autism, developmental delay, epilepsy, distinct facial features, craniofacial abnormalities, heart defects, immune dysfunctions, hyperactivity, and other systemic anomalies (Wetzel and Darbro, 2022). Notably, each microdeletion syndrome presents with a unique set of clinical manifestations.

20p12.3 microdeletion syndrome is a rare condition with a limited number of reported cases. The 20p12.3 region was initially implicated in human disease as early as 2001, when Leprêtre et al. (2001) first reported a small, interstitial 20p13p12.3 deletion in a patient with a severe growth deficit, providing early molecular characterization of gene loss within this genomic region. However, the condition was formally delineated as a distinct, recurrent genomic disorder, the 20p12.3 microdeletion syndrome (Lalani et al., 2009). Using a genotype-first approach, Lalani et al. identified multiple unrelated individuals with smaller, overlapping microdeletions specifically targeting 20p12.3. They established a causal link between this molecular change and a specific clinical triad, most notably Wolff–Parkinson–White (WPW) syndrome, along with variable developmental delay and characteristic facial dysmorphism. Furthermore, it is associated with various manifestations, including congenital hypopituitarism, growth hormone (GH) insufficiency, pituitary abnormalities, varying degrees of developmental delay, and facial dysmorphism (National Library of Medicine, 2024; Parsons et al., 2017).

This case report aims to contribute valuable insights to the understanding of 20p12.3 microdeletion syndrome. By comparing our case with similar cases reported worldwide, we aim to enhance the understanding of 20p12.3 microdeletion syndrome and provide valuable information for physicians in evaluating patients presenting with similar clinical features.

## Case report

A nine-year-old Saudi female presented to the pediatric genetics clinic in 2024 with dysmorphic features, growth faltering, resistance to GH therapy, and persistently low insulin-like growth factor 1 (IGF1) levels. She was the first daughter born to a healthy couple from the same tribe with no direct consanguinity, and had a healthy seven-year-old younger sister. A review of her neonatal history revealed that she was born early term at 37 weeks and 2 days via emergency C-section due to fetal bradycardia. At birth, she was jaundiced, and her weight and height were below the third percentile, although her head circumference was normal. She was admitted to the neonatal intensive care unit due to asymmetrical intrauterine growth restriction and was discharged after 4 days. According to her parents, she exhibited poor weight gain during her first year of life.

The patient reportedly experienced seizures, which are currently controlled using levetiracetam (250 mg). Her initial electroencephalogram in 2021 was abnormal, while the last one done in 2023 showed improvement along with unremarkable brain magnetic resonance imaging (MRI) findings. She also experienced recurrent episodes of ketotic hypoglycemia accompanied by high anion gap metabolic acidosis. However, during her most recent visit, she had remained free of hypoglycemic episodes for the past year. Her motor and social functions were normal compared to her developmental age at the time, and her parents reported no developmental complaints besides her short stature and growth faltering.

At the time of the visit, the patient weighed 15.30 kg (below the third percentile), had a height of 112.3 cm (below the third percentile), and had a head circumference of 50.5 cm (between the 10th and 25th percentiles). On examination, the patient exhibited malar hypoplasia, a high arched palate, microstomia, long philtrum, proptosis, and retrognathia. Her upper limbs exhibited hyperlaxity, clinodactyly of the fifth finger bilaterally, persistent fetal pads at the fingertips, and a single palmar crease on the right hand. Her lower limbs exhibited a sandal gap, shortening of the third toe bilaterally, mild nail hypoplasia in the lateral toes but severe hypoplasia in the third toe on the left foot, and bilateral coxa valga. The patient also showed scoliosis. Nonetheless, her physical examination was otherwise unremarkable. The patient’s most recent laboratory tests showed an IGF1 level of 151.1 ng/mL (49∼451 ng/mL), insulin-like growth factor binding protein (IGFBP) level of 3 ug/mL (1.8 ∼ 7.1 ng/mL), thyroid stimulating hormone (TSH) level of 3.7 mIU/L (0.7 ∼ 4.17 mlU/L), and free thyroxine (FT4) level of 13.1 pmol/L (11.4 ∼ 17.6 pmol/L).

The patient underwent three genetic investigations. In the first and second investigations, the initial analyses revealed no correlation between her genetic findings and the clinical presentation. Specifically, in the first genetic testing, whole-exome sequencing and methylation analysis were done to explore potential molecular abnormalities; however, both tests yielded negative results. Subsequently, in the second genetic testing, whole-genome sequencing (WGS) was conducted, but it also yielded negative findings, with no clinically significant or pathogenic variants detected that could explain the observed phenotype. In the third test, chromosomal microarray (CMA) and subsequent WGS analyses were performed with CMA confirmed a *de novo* heterozygous 3.5 Mb deletion spanning 20p13 to 20p12.3, termed 20p13p12.3 (chr20:4,781,649–8,358,774, human genome assembly GRCh37), encompassing 31 genes: *RASSF*, *SLC23A2*, *TMEM230*, *PCNA-AS1*, *PCNA*, *CDS2*, *PROKR2*, *LINC00658*, *LOC643406*, *LINC00654*, *LINC01729*, *GPCPD1*, *SHLD1*, *CHGB*, *TRMT6*, *MCM8*, *MCM8-AS1*, *CRLS1*, *LRRN4*, *FERMT1*, *CASC20*, *LINC01713*, *BMP2*, *LINC01428*, *LINC01751*, *LINC01706*, *MIR8062*, *HAO1*, *TMX4*, *PLCB1-IT1*, and *PLCB1*. Based on the gene content and overlap with previously described cases, this deletion was classified as likely pathogenic according to ClinGen and American College of Medical Genetics and Genomics (ACMG) guidelines, confirming the diagnosis of 20p12.3 microdeletion syndrome.

## Discussion

This case report described a nine-year-old female who presented with growth faltering, short stature, a well-controlled seizure disorder, metabolic disturbances, and distinctive dysmorphic facial features. Genetic analyses revealed a rare 3.5 Mb deletion spanning 20p13 to 20p12.3, consistent with 20p12.3 microdeletion syndrome, which is known to manifest with diverse symptoms and has been documented in only a limited number of case reports. Reported deletions involving this region have varied in size, typically ranging from 0.5 to 10 Mb (Table 1). The deletion identified in our patient lies near the middle of this range, which may partly explain the broad spectrum of endocrine, growth, and neurodevelopmental abnormalities observed, as well as the lack of certain features frequently reported in other cases. Another possible explanation for our patient’s phenotype relates to the specific genes encompassed within the deleted region. The 20p13p12.3 microdeletion identified in our patient encompasses 31 genes, several of which have not been previously reported in association with this chromosomal region. Among the genes within the deleted region, bone morphogenetic protein 2 (*BMP2*), hydroxyacid oxidase 1 (*HAO1*), thioredoxin-related transmembrane protein 4 (*TMX4*), phospholipase C beta 1 (*PLCB1*), and prokineticin receptor 2 (*PROKR2*) have been previously described in the literature and are known to play important roles in developmental, metabolic, and neuroregulatory processes.

**TABLE 1:** Clinical manifestations observed in our patient and 12 previously reported patients with 20p12.3 microdeletions.

Study		Deletion size & coordinates	Reported clinical manifestations
Our study		∼3.5 Mb GRCh37 4,781,649–8,358,774	Growth and development: Growth faltering, short stature, GH resistance, and persistently low IGF-1 levels Hand, feet, and MSK: Bilateral coxa valga, mild scoliosis, hyperlaxity of the upper limb, clinodactyly of the fifth finger bilaterally, shortening of the third toe bilaterally, persistent fetal pads, and palmar crease on the right hand Craniofacial: Malar hypoplasia, high arched palate, microstomia, long philtrum, proptosis, and retrognathia Cardiac and other: Seizures and ketotic hypoglycemic episodes with high AG metabolic acidosis, closed atrial septal defect
Leprêtre et al. (2001)		∼6 Mb YAC ∼2–8 Mb D20S842 to D20S900	Growth and development: *Growth faltering, short stature*, neurocognitive delay, motor delay, low IQ, psychomotor delay, and language deficits Hand, feet, and MSK: Bilateral club foot valgus Craniofacial: *Long philtrum*, micrognathia, hypertelorism, high forehead, wide alae nasi, DSPF, low-set ears, bulbous nose, depressed nasal bridge, anteverted nares, and epicanthal folds Other: Small penis with balanopreputial hypospadias and a thin upper lip
Lalani et al. (2009)	1 (M)	1.1 Mb ∼6–8 Mb	Growth and Development: *Growth faltering,* neurocognitive delay, and *short stature* Hand, Feet, and MSK: Pectus deformity and *persistent fetal pads* Craniofacial: *Malar hypoplasia*, *microstomia,* hypertelorism, DSPF, epicanthal folds, broad nasal root and bridge, maxillary hypoplasia, and macrocephaly Cardiac and Other: WPWS and *atrial septal defect*
	2 (M)	2.3 Mb ∼5–8 Mb	Growth and Development: Neurocognitive delay and motor delay Hand, Feet, and MSK: *Persistent fetal pads* and broad digits Craniofacial: *Microstomia, long philtrum,* hypertelorism, macrocephaly, DSPF, frontal upsweep, epicanthal folds, and small ears with thickened helices Cardiac and Other: WPWS.
	3 (F)	2.3 Mb ∼5–8 Mb	Growth and Development: *Short stature*, neurocognitive delay, and motor delay Hand, Feet, and MSK: *Persistent fetal pads* Craniofacial: *Malar hypoplasia*, hypertelorism, macrocephaly, and maxillary hypoplasia
	4 (F)	∼4.2 Mb ∼5–11 Mb	Growth and Development: Neurocognitive delay, motor delay, and *short stature* Hand, Feet, and MSK: Vertebral abnormalities Craniofacial: Hypertelorism, microcephaly, and maxillary hypoplasia Cardiac and Other: WPWS, iris coloboma, patent ductus arteriosus, pulmonic stenosis, PPAS, and hearing loss
	5 (F)	10.7 Mb ∼2–14 Mb	Growth and Development: *Short stature*, neurocognitive delay, and motor delay Hand, Feet, and MSK: Pectus deformity Craniofacial: Hypertelorism, DSPF, and cleft lip Cardiac and Other: WPWS, PPAS, hearing loss, jaundice, and right ventricular hypertrophy
Sahoo et al. (2011)	1 (F)	592.7 kb hg18 6,222,266–6,814,990	Growth and Development: *Growth faltering* Hand, Feet, and MSK: Sandal gap, pectus excavatum, diastasis recti, and short fifth finger Craniofacial: *Long philtrum*, cleft palate, micrognathia, glossoptosis, microcephaly, and large communicating fontanelles Other: Vertical creases, deep palmar flexion creases, and patent foramen ovale
	2 (F)	566.7 kb hg18 6,265,253–6,831,640	Growth and Development: *Growth faltering and* language deficits Hand, Feet, and MSK: Zygodactylous triradius between the second and third right toes Craniofacial: *Microstomia, long philtrum*, cleft palate, bifid uvula, microcephaly, large eyes, synophrys, anteverted nares, cholesteatoma, and *high arched palate* Other: Hearing loss
	3 (F)	5.37 Mb hg18 672,605–9,042,183	Growth and Development*: Growth faltering*, motor deficits with head lag and wobble, motor deficits with head lag and wobble, feeding difficulties, and developmental hip dysplasia Hand, Feet, and MSK: Hyperextensibility of hands and feet, recurrent hip disclocation, and decreased muscle mass Craniofacial: *Long philtrum*, DSPF, micrognathia, cleft palate, flat facial profile, depressed nasal bridge, anteverted nares, pinpoint hemeangioma on the tip of the nose, prominent forehead, and transverse crease across the chin Other: Central hypotonia and hyporeflexia
Williams et al. (2012)		∼2.3 Mb hg18 6,265,251–8,523,663	Growth and Development: *Growth faltering,* psychomotor delay, and self-stimulatory behaviors Hand, Feet, and MSK: Digital hypoplasia between the second and third toes, and mild dextroscoliosis Craniofacial: *Long philtrum*, micrognathia, cleft palate, bifid uvula, microcephaly, widened palpebral fissure, and pronounced central incisors
Amasdl et al. (2016)		N/A	Growth and Development: *Growth faltering* and psychomotor delay Craniofacial: *Microstomia*, short philtrum, micrognathia, small forehead, hypertelorism, DSPF, low-set ears, broad nasal bridge, bulbous nose, downturned corners of the mouth, widespread tooth decay, dental overlapping, cleft palate, and bifid uvula
Parsons et al. (2017)		∼4.8 Mb GRCh37 5,281,784–10,070,297	Growth and Development: *Growth faltering* Craniofacial: *Long philtrum*, micrognathia, hypoplastic anterior lobe and ectopic posterior lobe, *malar hypoplasia*, flat facial profile, depressed nasal bridge, and anteverted nares Other: Conjugated hyperbilirubinemia and hypoglycemic episodes with seizures

Abbreviations: N/A, not available; AG, anion gap; DSPF, down-slanting palpebral fissures; F, female; GH, growth hormone; M, male; MSK, musculoskeletal; PPAS, peripheral pulmonary artery stenosis; WPWS, Wolff–Parkinson–White syndrome; YAC, yeast artificial chromosome.

* Manifestations that are present in our patient are italicized for other cases.

* Manifestations that are unique to our patient are underlined in the “Our Study” row.

* Lepetre et al. (2001) used an old genome build (YAC), with the deletion spanning microsatellite markers D20S842 to D20S900.

The patient presented in this case report was diagnosed with a seizure disorder, which has been effectively controlled using levetiracetam. Notably, seizures are exceptionally rare in individuals with 20p12.3 microdeletion syndrome, having only been described in a single published case involving a five-year-old girl. In that patient, the seizures were temporally associated with episodes of hypoglycemia during the neonatal period and resolved within the first 2 weeks after birth, suggesting a transient, metabolically driven etiology rather than a persistent epileptic disorder (Parsons et al., 2017).

Notably, one gene in the deleted region in our patient, *PROKR2*, has been implicated as a potential contributor to seizure susceptibility, as pathogenic variants in this gene have been associated with epileptic manifestations. *PROKR2* functions in the production of a receptor crucial for embryonic gonadotropin-releasing hormone and olfactory neuron migration, which, when affected, disrupts overall pituitary development. This disruption leads to neuroendocrine disorders, including GH deficiency, multiple pituitary hormone deficiency, and, in some severe cases, seizures (Kardelen et al., 2023).

In two previously reported cases, patients with mutations in *PROKR2* presented with seizures that were temporally associated with episodes of hypoglycemia (Parsons et al., 2017; Reynaud et al., 2012). Both of these cases shared several features, including pituitary abnormalities and seizure activity directly linked to hypoglycemic events. However, a notable distinction in our patient is the finding of a normal brain MRI and the lack of a temporal association between hypoglycemia and seizure episodes despite the presence of recurrent hypoglycemia. It suggests that hypoglycemia was unlikely to be the direct cause of our patient’s seizures and that additional pathophysiologic mechanisms may underlie seizure susceptibility in 20p12.3 microdeletion syndrome beyond metabolic disturbances alone. Moreover, it is clinically relevant, as seizures that coincide temporally with hypoglycemia are generally considered acute symptomatic events resulting from neuroglycopenia, whereas seizures occurring independently suggest an alternative underlying mechanism (Bartolini et al., 2023).

Similarly, *PLCB1*, which encodes a phosphoinositide-specific enzyme, plays a crucial role in neuronal signaling, with its loss disrupting inhibitory neuronal circuits, leading to increased neuronal excitability and a lower seizure threshold (Ngoh et al., 2014), which could also account for the seizures observed in our patient.

Additionally, 20p12.3 microdeletion syndrome has been associated with various cardiac abnormalities. Lalani et al. (2009) established a link between 20p12.3 microdeletion syndrome and WPW syndrome. However, after thorough evaluation, our patient was found not to have WPW syndrome. Nonetheless, other cardiac abnormalities have been reported in association with 20p12.3 microdeletion syndrome, including atrial septal defect, which was present in our patient and had also been reported by Lalani et al. (2009). Additionally, patients with 20p12.3 microdeletion syndrome have been documented to have patent ductus arteriosus, patent foramen oval, pulmonic stenosis, peripheral pulmonary artery stenosis, and right ventricular hypertrophy (Lalani et al., 2009; Sahoo et al., 2011; Williams et al., 2012). However, almost half of the patients with 20p12.3 microdeletion syndrome reported in the literature (8/12 [66.6%]) did not present with cardiovascular abnormalities. The cardiac abnormalities observed in 20p12.3 microdeletion syndrome could be attributed to deletion of *BMP2*, which has been strongly implicated in cardiac development and conduction defects. *BMP2* plays a crucial role during embryogenesis, particularly in the formation of the cardiac valve septa and outflow tract. Disruption of *BMP2* function has been associated with structural and functional cardiac abnormalities (Tan et al., 2017).

Our patient presented with growth faltering, consistent with what has been reported in the literature, where 9/12 (75%) patients with 20.12.3 microdeletion syndrome exhibited growth failure (Table 1). In a case report by Parsons et al. (2017), the patients exhibited GH deficiency and were treated with recombinant human GH, to which they had a positive response, resulting in a notable increase in height velocity to 8.8 cm per year. In contrast, our patient did not respond to GH therapy. At her last visit to the endocrine department, she had a height of 112.3 cm (*z*-score = −5.26), a weight of 15.2 kg (*z*-score = −3.8), and a growth velocity of 8 cm/4 months, indicating that her growth was below average for her age. Our patient’s growth faltering and lack of response to exogenous GH therapy are indicative of GH insensitivity (GHI), likely caused by the loss of one or more genes in the identified 20p13p12.3 microdeletion. For instance, the deletion of *BMP2* will directly disrupt skeletal and growth plate development, as the encoded protein is critical in bone, cartilage, and growth plate formation. Indeed, haploinsufficiency for *BMP2* compromises the structural integrity of the growth plates, rendering them inherently resistant to GH and its mediator, IGF1, regardless of GH levels. Thus, it represents a form of GHI at the level of the target tissue itself (Tan et al., 2017).

**FIGURE 1:**
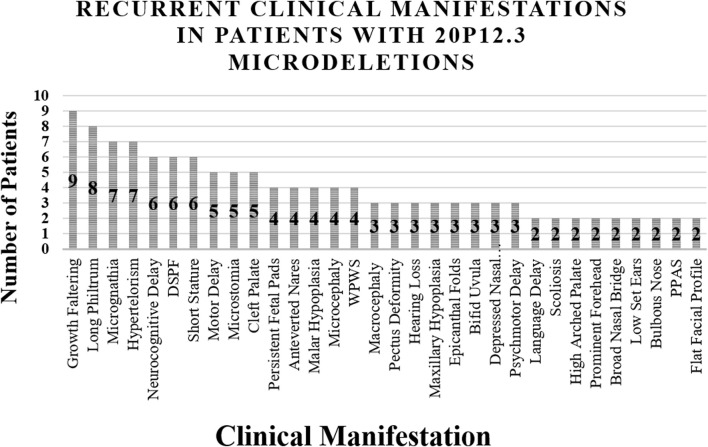
Major Clinical Findings Presented in Patients with 20p12.3 Microdeletions. Abbreviations: DSPF = down-slanting palpebral fissures, WPWS = Wolff–Parkinson–White syndrome, PPAS = peripheral pulmonary artery stenosis.

The clinical manifestations observed in reported cases of 20p12.3 microdeletion syndrome are summarized in Table 1 and Figure 1. Our patient exhibited syndromic features, including malar hypoplasia, consistent with 3/12 (25.0%) previously reported cases. She also presented with a high-arched palate, a feature described in 1/12 (8.3%) previously reported cases. In contrast, she presented with proptosis, which has not been previously reported in cases of 20p12.3 microdeletion syndrome, making it a novel finding in our patient. Our patient also presented with microstomia, which has also been reported in 3/12 (25.0%) previously reported cases. Among the recurrent manifestations, growth faltering (*n* = 10/12, 83.3%) and a long philtrum (*n* = 9/12, 75.0%) were the most frequently documented features.

Interestingly, Amasdl et al. (2016) described a patient with a short philtrum, emphasizing the phenotypic variability among patients with 20p12.3 microdeletion syndrome. While micrognathia was reported in 7/12 (58.3%) previously reported cases, our patient exhibited retrognathia. Williams et al. (2012) described a patient with microretrognathia, a finding that bridges the features observed in our patient and those reported previously. Other findings, such as hypertelorism (*n* = 7/12, 58.3%) and neurocognitive delay (*n* = 6/12, 50.0%), were not present in our patient. Additional recurrent features of patients with 20p12.3 microdeletion syndrome include down-slanting palpebral fissures (*n* = 5/12, 41.7%), cleft palate (*n* = 5/12, 41.7%), microcephaly (*n* = 4/12, 33.3%), WPW syndrome (*n* = 4/12, 33.3%), motor delay (*n* = 5/12, 41.7%), and a flat facial profile (*n* = 2/12, 16.7%). Persistent fetal pads on the hands were the only recurrent upper-limb finding, reported in three cases by Lalani et al. (2009) and in our patient. Lower-limb manifestations, including subtle hyperextensibility of the hands and feet and a zygodactylous triradius between the second and third right toes, were described in single cases. Additional skeletal findings included pectus deformity and vertebral anomalies.

In conclusion, this case report contributes to the understanding of 20p12.3 microdeletion syndrome, highlighting its clinical heterogeneity and genetic complexity. Our patient presented with novel features, including episodes of ketotic hypoglycemia with high anion gap metabolic acidosis, GHI, persistently low IGF1 levels, malar hypoplasia, hyperlaxity of the upper limbs, clinodactyly of the fifth fingers bilaterally, a single palmar crease on the right hand, shortening of the third toes bilaterally, and severe hypoplasia of the left third toe. Together with previously reported features, these findings illustrate the broad phenotypic spectrum associated with 20p12.3 microdeletion syndrome. The variability in phenotype likely reflects variation in the size and genomic location of the deletion, reinforcing the importance of comprehensive clinical evaluation. This case report also emphasizes the critical role of CMA and WGS in establishing a definitive diagnosis, refining genotype–phenotype correlations, and guiding genetic counseling and management.

## Data Availability

This article includes the data that supports the case report’s conclusions. Further information cannot be made public due to patient confidentiality and privacy concerns. Upon reasonable request and with the necessary ethical approvals, the corresponding author may provide more information.
